# The cost of beauty: Perspectives of salon workers in Kisumu City, Kenya

**DOI:** 10.1371/journal.pgph.0002503

**Published:** 2023-11-06

**Authors:** Patrick Ogola Onyango

**Affiliations:** Department of Zoology, Maseno University, Kisumu, Kenya; PLOS: Public Library of Science, UNITED STATES

## Abstract

Despite occupational exposure to hazardous chemicals in cosmetics and personal care products (PCPs), salon workers receive minimal formal protections. Consequently, most salon workers rely on personal safeguards. However, the nature of such individual-level safeguards remains unknown. Knowledge of risks associated with occupational use of cosmetics and PCPs and information needs of salon workers were investigated in Kisumu City, Kenya. Responses from 302 respondents showed that 84% were women and 30% had post-secondary education. Seventy percent reported knowing that ingestion, inhalation, dermal absorption, and injection are the pathways through which harmful products in cosmetics and PCPs may enter the body. Salon workers who had been employed for more than 5 years were at least twice more likely to report that it is not the case that cosmetics and PCPs only cause harm to children (5–10 years vs 1 year: OR = 2.440, 95% CI, 1.160–5.239; >10 years vs 1 year: OR = 8.857, 95% CI, 3.163–29.377); they were about three times more likely to either agree with the statement that cosmetics and PCPs only cause harm under prolonged exposure or to say that they did not know compared to their counterparts who had worked in the industry for 1 year (5–10 years vs 1 year: OR = 2.750, 95% CI, 1.144–7.179; >10 years vs 1 year: 3.179, 95% CI, 1.173–9.096). Over 50% of the respondents reported that they need information on how to protect themselves and others; on available protective measures; and on cosmetic products and PCPs that are safe. Sixty percent reported that they would prefer to get such information from the Ministry of Health at the county or national level and on product inserts. Overall, salon workers in Kisumu City are knowledgeable about the risks associated with their occupation but also appreciate gaps in their knowledge, which can be filled by government-mandated interventions.

## Introduction

Cosmetics and personal care products (PCPs) represent a pernicious source of endocrine-disrupting chemicals, carcinogens, reproductive toxicants, and respiratory and skin irritants. All these chemicals are associated with adverse effects. Beyond irritation to the eye, nose, throat and skin during or following use of cosmetics and PCPs, women salon workers are at increased risk of having infants with birth defects such as anotia, microtia, and cleft palate [1, 2]; reproductive disorders [3], and negative pregnancy outcomes [4]; maternal complications such as gestational diabetes [5]. In addition, workplace exposure to products in cosmetics and PCPs has been associated with cancer [6].

The adverse health effects associated with occupational exposure to cosmetics and PCPs are attributed to a diverse range of chemicals of concern including known classes of endocrine-disrupting chemicals such as parabens and phthalates [7, 8], carcinogens and mutagens such as polycyclic aromatic hydrocarbons (PAHs) [9], reproductive toxicants such as PAHs [9], and respiratory and skin irritants such as formaldehyde and toluene [10].

In spite of the occupational risks, salon workers lack formal protections and have limited opportunities for recourse. Yet across the globe, salon workers are often a vulnerable group because they are more likely to be immigrants [11], and people of low socio-economic and education status [11, 12]. In the absence of formal protections, they tend to work under poor working conditions that expose them to ergonomic complications and both physical and psychological abuse [13–15], experiences that may exacerbate consequences of exposure to hazardous chemicals in cosmetics and PCPs.

Many countries lack or have weak or poorly policed regulations for the manufacture, distribution and use of cosmetics and PCPs [16]. In Kenya, for example, beyond the definition of cosmetics in the Food, Drugs and Chemical Substances Act of 2012 [17], there is a general lack of standards on the manufacture, distribution and use of cosmetics. In many countries, therefore, individual-level knowledge and safeguards remain the only protection or mitigation against workplace exposure to cosmetics and PCPs. However, there is a paucity of data on risks associated with occupational exposure to cosmetics and PCPs and on the nature of such individual-level safeguards, if they exist, and how they are patterned among salon workers. In the absence of knowledge on safety and mandated protections, the adverse environments in which salon workers operate increases risk of harm to both the salon workers as well as their clients thus presenting a substantial public health problem.

Knowledge of risks associated with workplace exposures to chemicals is a vital component in efforts to maximize occupational safety of salon workers. For instance, previous studies on knowledge of risks associated with occupational exposure to chemicals contained in cosmetics and PCPs among salon workers have focused on the role of training on knowledge of safety measures in salons [18]. For example, Quach et al. [18] showed that salon workers who were trained demonstrated improved knowledge of safety precautions in salons and were more likely to use protective measures such as use of protective clothing. In the State of Michigan, for instance, Le et al. [19] found evidence that suggested the need for continuing education for nail salon workers. However, the knowledge base of salon workers in countries such as Kenya that lack national guidelines on both occupational safety for salon workers and mandatory training is unavailable. The present study investigated knowledge of risks associated with occupational exposure to cosmetics and PCPs and information needs of salon workers in Kisumu City, Kenya.

## Methods

The study targeted salon workers in Kisumu City, located in western Kenya. The public health problem of occupational safety in salons is widespread in many towns including Kisumu. The unique aspect to the problem that Kisumu presents is the near-proximity between low-end and seemingly high-end salons. It was hoped that this attribute in diversity would enrich our understanding of occupational safety from first-hand accounts of salon workers. As is the case in most urban areas, the number of salons in the city has grown quite rapidly. In a preliminary study, it was estimated that the city had 330 salons with 951 salon workers. Yamane (1967) sample size calculation formula [20] was used to select a sample of 310 salon workers after a 10% adjustment.

For sampling purposes, the city was divided into five sampling sections: Central, Eastern, Northern, Southern, and Western each having 427, 332, 63, 72 and 57 total number of salon workers respectively. Probability proportion to size was employed so as to cater for the variability in the distribution of number of salon workers across the five regions. This resulted in targeted sample size of 139, 108, 21, 23, and 19 for Central, Eastern, Northern, Southern, and Western regions respectively. Thereafter, simple random sampling was used to select salons. In each salon, we recruited workers who consented to participate in the study.

Ethical approval for the present study was granted by the Maseno University Ethics Review Committee, Ref: MSU/DRPI/MUERC/00694/19. Written consent was obtained from all study participants.

### Data collection

A semi-structured questionnaire that was available in three languages commonly spoken in the study area, Dholuo, English and Kiswahili, was used for data collection. The questionnaire had six sections dealing with demographic information, knowledge of risks associated with occupational exposure to cosmetics and PCPs, risk perception, intention to use protective measures, motivating or hindering factors for use of protective measures, and information needs. Data on intention to carry out protective measures and motivating or hindering factors for use of protective measures are not presented here. For each duly consented respondent, data were collected on socio-demographic characteristics of respondents and on potential harm associated with workplace exposure to cosmetics and PCPs. For potential harm associated with exposure to cosmetics and PCPs, respondents were asked about their knowledge of how any potentially hazardous chemicals contained in cosmetics and PCPs may enter the body as well as their knowledge of risks associated with exposure to cosmetics and PCPs. Lastly, respondents were asked questions to gauge their information needs. Specifically, the questions in this section of the questionnaire dealt with issues such as the type of information needed, if any, and how they preferred the information to be communicated to them. The questionnaire was pre-tested on a population of salon workers in Ahero Town, located about 20 km east of Kisumu City. Pre-testing the questionnaire enabled us to revise any questions that were not clear. Administration of each questionnaire took 20–45 min.

In the main study, we targeted one employee per salon. However, there were instances where we administered the questionnaire to more than one salon worker where more than one in a salon volunteered to participate in the study; this enabled us to improve response rate given that some salon workers were not willing to participate in the study for several reasons including need for employer’s permission but the employer was not present when our team visited the salon; monetary compensation and lack of time to take part in the survey. In such where more than one salon worker in a given salon volunteered to participate in the study, we made sure to separate the respondents so as to ensure privacy and to reduce chances of one hearing responses given by their colleague.

### Data analysis

Data on socio-demographic characteristics and knowledge were summarized using counts and percentages. Chi-square goodness-of-fit test was used to determine whether frequencies were randomly distributed; Chi-square test of independence was used to determine association between socio-demographic characteristics of salon workers and their knowledge of adverse effects associated with exposure to cosmetics and PCPs. Fisher’s exact test was applied in cases where expected frequencies were less than 5. Statistically significant associations were further analyzed using logistic regression using the glm function with a binomial family. Threshold for statistical significance was set at ≤ 0.05. All statistical analyses were conducted in R 4.2.1.

## Results

### Socio-demographic characteristics of study participants

Table 1 summarizes the socio-demographic characteristics of study participants. In brief, a majority of the respondents were women (n = 253, 84%) and individuals aged 18–49 years (94%), slightly more than a half (56%) were married, and a majority (63%) had children. In terms of level of education, nearly 70% of the respondents had secondary level of education and below. In terms of how easy it is to find employment in salons, the responses were as follows: Easy = 52 (17.3%); Somewhat easy = 90 (30.0%); Somewhat difficult = 130 (43%); difficult = 28 (9.3%).

**Table 1:** Socio-demographic characteristics of salon employees in Kisumu City, Kenya.

Characteristic	Frequency (n)	Percentage (%)
**Gender**		
Female	253	83.8
Male	49	16.2
**Age**		
18–49 Years	284	94.4
49 years and older	17	5.6
**Level of education**		
No formal education	2	0.7
Primary	36	12.1
Secondary	170	57.0
Tertiary	77	25.8
University	13	4.4
**Marital status**		
Single	118	39.3
Married	168	56.0
Widowed	6	2.0
Separated	5	1.7
Divorced	3	1.0
**Have children**		
Yes	189	63.0
No	111	37.0
**Ease of finding employment in salons**		
Easy	52	17.3
Somewhat Easy	90	30.0
Somewhat Difficult	130	43.3
Difficult	28	9.3
**Years in employment**		
Less than 1 Year	53	18.0
>1<5 Years	137	46.4
5–10 Years	68	23.1
>10 Year	37	12.5

### Knowledge of risks associated with exposure to cosmetics and personal care products

At least 70% of respondents indicated that they were knowledgeable about the pathways through which any toxic chemicals contained in cosmetics and PCPs may enter the body; Table 2. On the question of whether cosmetics and PCPs can cause harm, a significant majority of the respondents reported that they knew that there is potential harm associated with use of these products (Chi-square Goodness-of-fit test, p = 0.000), knowledge of available protective measures (p = 0.000), and that it is not the case that toxic chemicals in these products only cause harm to children (p = 0.000). Surprisingly, 66% of respondents reported that exposure to these products only causes harm under prolonged periods of exposure (p = 0.000), Table 2.

**Table 2:** Knowledge of risks associated with exposure to cosmetics and personal care products.

Knowledge question	Correct [n, (%)]	Incorrect [n, (%)]	Don’t know [n, (%)]	*χ*2 statistic	P value
***Mode of entry of chemicals contained in cosmetics and PCPs***					
Ingestion	247(84.6)	14(4.8)	31(10.6)	364.69	0.000
Inhalation	243(81.8)	30(10.1)	24(8.1)	314.36	0.000
Skin absorption	242(81.8)	29(9.8)	25(8.4)	312.41	0.000
Injection	214(74.0)	30(10.4)	45(15.6)	216.75	0.000
***Risks associated with use of cosmetics and PCPs***					
Exposure can cause ill health	260(87.8)	21(7.1)	15(5.1)	395.89	0.000
There are protective measures that I can take	286(95.7)	6(2.0)	7(2.3)	522.55	0.000
Cosmetics and PCPs only cause harm to children	90(31.4)	166(57.8)	31(10.8)	95.76	0.000
Cosmetics and PCPs only cause harm under prolonged exposure	195(65.9)	75(25.3)	26(8.8)	153.25	0.000

Only period of employment was associated with knowledge of risks associated with occupational use of cosmetics and PCPs; Table 3. Specifically, there was a significant association between period of employment and the respondents answers to the statements “*Cosmetics and PCPs only cause harm to children”* and “*Cosmetics and PCPs only cause harm under prolonged exposure”*. Further analysis using logistic regression showed that salon workers who had been employed for more than 5 years were at least twice more likely to report that it is not the case that *cosmetics and PCPs* only cause harm to children. In addition, they were three times more likely to either agree with the statement that cosmetics and PCPs only cause harm under prolonged exposure or to say that they did not know compared to their counterparts who had been employed for less than 1 year.

**Table 3:** Association between socio-demographic characteristics and knowledge of risks from cosmetics and personal care products.

Characteristic and knowledge of risk	*χ*2 or Z statistic	P value	OR (95% CI)
**Gender**			
Exposure to cosmetics can cause harm	0.163	0.686	**-**
There are protective measures I can take when using cosmetic products and PCPs	1.446	0.229	**-**
Cosmetics and PCPs only cause harm to children	0.955	0.329	**-**
Cosmetics and PCPs only cause harm under prolonged exposure	2.887	0.089	**-**
**Age**			
Exposure to cosmetics can cause harm	0.192	0.661	**-**
There are protective measures I can take when using cosmetic products and PCPs	0.087	0.768	**-**
Cosmetics and PCPs only cause harm to children	1.232	0.267	**-**
Cosmetics and PCPs only cause harm under prolonged exposure	2.361	0.124	**-**
**Education level**			
Exposure to cosmetics can cause harm	3.023	0.554	**-**
There are protective measures I can take when using cosmetic products and PCPs	2.623	0.622	**-**
Cosmetics and PCPs only cause harm to children	8.191	0.085	**-**
Cosmetics and PCPs only cause harm under prolonged exposure	6.170	0.187	**-**
**Marital Status**			
Exposure to cosmetics can cause harm		0.735	**-**
There are protective measures I can take when using cosmetic products and PCPs		0.773	**-**
Cosmetics and PCPs only cause harm to children		0.432	**-**
Cosmetics and PCPs only cause harm under prolonged exposure		0.604	**-**
**Period in employment**			
Exposure to cosmetics can cause harm	5.386	0.146	**-**
There are protective measures I can take when using cosmetic products and PCPs	5.242	0.155	**-**
Cosmetics and PCPs only cause harm to children	19.450	**0.000**	
< 1 Year	Ref	Ref	Ref
>1 < 5 Years	1.438	0.151	1.619 (0.843–3.152)
5–10 Years	2.327	**0.020**	2.440 (1.160–5.239)
>10 Years	3.897	**0.000**	8.857 (3.163–29.377)
Cosmetics and PCPs only cause harm under prolonged exposure	9.001	**0.029**	
< 1 Year	Ref	Ref	Ref
>1 < 5 Years	0.817	0.414	1.433 (0.627–3.594)
5–10 Years	2.182	**0.029**	2.750 (1.144–7.179)
>10 Years	2.236	**0.025**	3.179 (1.173–9.096)
**Having children**			
Exposure to cosmetics can cause harm	0.132	0.716	**-**
There are protective measures I can take when using cosmetic products and PCPs	0.634	0.426	**-**
Cosmetics and PCPs only cause harm to children	0.074	0.785	**-**
Cosmetics and PCPs only cause harm under prolonged exposure	1.350	0.245	**-**

*χ*2 values are reported for chi-square tests except for Fisher’s exact test; Z statistic is reported for logistic regression.

### Information needs of salon workers

In terms of information needs, over 50% of the respondents reported that they needed information on how to protect themselves from exposure to any harmful chemicals contained in cosmetics and PCPs, how to protect others, available protective measures against toxic chemicals contained in cosmetics and PCPs, and on cosmetic products that are safe. Regarding how they preferred the information to be communicated, about 60% reported that they prefer to get the information from the Ministry of Health at the county or national level and on product inserts, Table 4.

**Table 4:** Information needs of salon workers in Kisumu City, Kenya.

What are the most important topics for which you would like to receive information at this time? [n = 294]	Frequency (%)
How to protect yourself from exposure to cosmetic products	79.9
How to protect others from exposure to cosmetic products	54.1
What protective measures are available for those using cosmetic products	68.0
Which cosmetic products are safe	57.1
What to do when you are exposed cosmetic products known to cause ill health	41.8
Symptoms of ill health from exposure to cosmetic products	29.9
**Who would you like to provide you with this information? [n = 295]**	
General practitioner	55.9
County Public Health Service	68.5
National authorities (for example, Ministry of Health)	68.5
**How would you like to receive this information? [n = 297]**	
Information meeting by the Ministry of Health	58.6
Leaflets from the County Government	37.7
Leaflets from the National Government	32.0
Information in local newspapers	43.4
Details on product inserts	57.6
Information on local radio stations	32.7

## Discussion

Socio-demographic characteristics of salon workers in Kisumu City are comparable to those reported for other countries in demonstrating that occupational cosmetology is dominated by women, individuals of reproductive age as well as those with relatively low education level [11, 21]. Additionally, the relatively short period of employment among salon workers in Kisumu City may point to both negative effects of exposure to cosmetics and PCPs and ergonomic complications associated with salon work [13]. For example, salon workers are required to be on their feet for long periods of time especially over the weekends. The consequence is that older salon workers, who may not be able to withstand the physical strain associated with salon work, may be retiring to less stressful employment. Alternatively, given the occupational hazards associated with handling products that have been associated with cancers, it could be the case that some of the workers die earlier than would be expected by chance.

### Knowledge of risks associated with occupational use of cosmetics and personal care products

A majority (70%) of the salon workers were knowledgeable about pathways through which any toxic substances in cosmetics and PCPs enter the body. This finding is consistent, in a general cosmetological context, with that of a study conducted in Uganda on nail cosmeticians [12], which showed that a majority of nail workers in Kampala City were knowledgeable about potential harms from hazardous materials contained in nail care products.

In terms of risks associated with occupational exposure to cosmetics and PCPs, a majority (88%) of the salon workers were aware that occupational exposure to cosmetics and PCPs can cause ill health. Previous studies that have reported comparably high levels of knowledge have attributed it to workplace training of salon workers [12, 18]. In this study, the role of workplace training on knowledge of risks associated with occupational exposure to cosmetics and PCPs was not investigated. However, it can be argued that in the context of the weak regulatory framework for cosmetics and PCPs in most developing countries including Kenya, salon workers hardly get any workplace training. Consequently, the relatively high level of knowledge reported here most likely derives from experiences of salon workers. Indeed, the relatively high level of knowledge is not surprising given several immediate and observable effects, including eye and skin irritation and respiratory distress, associated with exposure to cosmetics such as nail polish and nail polish removers. Nail care products such as polish and polish removers and enamel are known to have strong smells that irritate the eyes, throat, and skin [7, 22]. Some of these effects are so severe. For example, a study done in Michigan, USA, reported the case of a salon worker who was unable to report to work due to hypersensitivity but that these symptoms were abated when they stopped working at their salon [16].

The role of lived experiences may also explain, at least for salon workers with several years of work experience, the association between period of employment and knowledge of risks associated with exposure to cosmetics and PCPs. It is interesting to note that period of employment accounted for differences in the knowledge of risks associated with occupational exposures to cosmetics and PCPs given direct and immediate effects of some cosmetics and PCPs such as those that irritate the eye, throat and skin. Although there may be several explanations for the role of period of employment, it is plausible that salon workers, especially those who have not been in the business for a long time, over-discount, down-play or blame themselves for adverse effects of occupational exposure to cosmetics and PCPs. Self-blame, more specifically, has been demonstrated to be a coping mechanism among individuals who are occupationally exposed to hazardous chemicals or those who experience abuse.

### Information needs of salon workers

Responses from a majority of salon workers suggests that they were concerned about how they can protect themselves, how to protect others, and which cosmetic products are safe. These areas of information need likely highlight the workers’ appreciation of risks and harm associated with exposure to cosmetics and PCPs. In contrast, the topic that was reportedly of least importance to the salon workers was information on symptoms of ill health from exposure to cosmetic products. A likely interpretation of this result is that most of the salon workers have at one point or another experienced or witnessed harmful effects of cosmetics and PCPs. This interpretation supports the argument that salon workers may be down-playing harm associated with occupational exposure to cosmetics and PCPs or blaming themselves for such harm.

On the question of the preferred medium of communication or source of information, the respondents reported that they would prefer information to be provided to them by the Ministry of Health as well as in product inserts. The two preferred sources of information point to two key issues. First, asking to get information from the Ministry of Health officials either at the county or national level suggest a sense of trust or authority that salon workers place on the ministry in addition to or alternatively, the need for government-mandated protection of the salon work or cosmetic industry more generally. Second, it appears that most products do not have information on ingredients ergo denying salon workers information on any harmful effects of exposure as well as precautions that they need to take when using these products.

Taken together, the strength of this study is that it provides first-hand account on perspectives of salon workers on the potential hazards of their occupation. In so doing, it makes a strong case for much needed formal interventions to safeguard health and occupational safety of salon workers. However, as is the case with cross-sectional studies, it suffers two potential weaknesses. First, the constraint of self-reports is social desirability bias in which case respondents may have either concealed or exaggerated their responses. Second, the study did not collect data on whether the study participants were trained and the nature of such training. In the absence of such data, we can only speculate that the source of knowledge on risks associated with occupational exposure is experiential.

## Conclusions

Two major themes emerged from the present study. First, salon workers in Kisumu City demonstrated a relatively high level of knowledge of risks associated with exposure to cosmetics and PCPs. The high level of knowledge is likely informed by the workers experiences with exposure to these products. Second, they have an appreciation of the kinds of information that they need and that the Ministry of Health and inserts in the products are the preferred ways for communicating such information. In sum, this study concludes that the individual-level safeguards may not be adequate for occupational safety of salon workers. Consequently, government-mandated interventions are needed. Such interventions can take the form of policy frameworks training and certification.

## Supporting information

S1 Data S1 Data details the set of data that has been used in the manuscript. The coding and labels for columns are quite intuitive when read in conjunction with the S1 Questionnaire.
(XLSX)

S1 File The survey tool is a semi-structured questionnaire that was available in three languages commonly spoken in the study area, Dholuo, English and Kiswahili. The questionnaire had six sections focused on demographic information, knowledge of risks associated with occupational exposure to cosmetics and PCPs, risk perception, intention to use protective measures, motivating or hindering factors for use of protective measures, and information needs.
(PDF)
