# Supplementary material for: The cost of beauty: Perspectives of salon workers in Kisumu City, Kenya
Source: PLOS Glob Public Health. 2023 Nov 6;3(11):e0002503. doi: 10.1371/journal.pgph.0002503 (PMC10627437; doi:10.1371/journal.pgph.0002503)
Supplement: S1 File — The questionnaire had six sections focused on demographic information, knowledge of risks associated with occupational exposure to cosmetics and PCPs, risk perception, intention to use protective measures, motivating or hindering factors for use of protective measures, and information needs. (PDF) [file pgph.0002503.s002.pdf]

CONSENT FORM

Study title: Individual and occupational user perspectives on cosmetic and personal care products

PI Name: Dr. Patrick O. Onyango, Maseno University

Study Location: Kisumu Town

Introduction

Hello. My name is _______________________. I am working on a survey on perspectives on personal care products among individuals employed in beauty parlors. We would very much appreciate your participation in the survey.

Purpose of research project

We are carrying out the research survey to enable us better understand the knowledge, risk perception, and predictors of use of protective measures by employees in beauty parlors.

Voluntary participation

Your participation in the survey is voluntary. You can choose not to answer any or all the questions if you are not comfortable. You may withdraw or stop your participation in the survey at any time for any reason. There is no consequence for stopping to participate in the survey.

Why you are being asked to participate

You are being asked to participate in the survey because you are employed in a beauty parlor where you handle cosmetics and personal care products. Your responses will enable us to understand the knowledge, risk perception, and predictors of use of protective measures by employees in beauty parlors.

Procedures

If you agree to participate in the survey, we will ask you questions on the following topics:

1. Your demographic details
2. Your knowledge of personal care products
3. Your perception of risks associated with personal care productions
4. What would make you use or not use protective measures when using personal care products

Risks

There are no significant risks associated with your participation in the survey. We will do our best to keep all information that you provide us in this survey confidential.

Benefits

There are no direct benefits, monetary or otherwise, associated with your participation in the survey. However, it is anticipated that participants will gain future benefits should there be interventions or policy changes as a result of the research project. A respondent who is concerned that exposure to cosmetics and personal care products have negatively impact their health will be referred for medical attention.

Confidentiality

All information you provide us during the survey will be stored confidentially. In order to make sure that your privacy is safeguarded, we will not reveal your personal details such as your name who is not part of this study.

Do you have any questions?

Who do you call if you have any questions or problems?

You can contact **Dr. Patrick Onyango at 0725-039-577**

You can also call the **Secretary of the Maseno University Ethics Review Committee**, Private bag, Maseno; Telephone numbers: 057-51622, 0722203411, 0721543976, 0733230878; Email address: muerc-secretariate@maseno.ac.ke; muerc-secretariate@gmail.com

What does your written consent mean?
Your written consent means:

1. You have been informed about this survey's purpose, procedures, possible benefits and risks.
2. You have been given the chance to ask questions about the survey.
3. You have voluntarily agreed to participate in the survey.

Individual consent to participate in the survey ☐

____________________	__________
Signature or thumb print	Date

____________________	____________________	__________
Print name of person obtaining consent	Signature of person obtaining consent	Date

Give a copy of the signed consent form to the participant and keep one copy in study records.
End of consenting process.

QUESTIONNAIRE FOR BEAUTY PARLOR EMPLOYEES
Part A: DEMOGRAPHIC INFORMATION

Name of salon____

Please select

1. Gender
   - Female ☐
   - Male ☐
2. Age of respondent

Age in Years	Answer
18-49	
Above 49	

3. Level of education
   - Primary ☐
   - Secondary ☐
   - Tertiary ☐
   - University ☐
   - Never attended ☐
4. Marital status
   - Single ☐
   - Married ☐
   - Widowed ☐
   - Separated ☐
   - Divorced ☐
5. Do you have any children?
   - YES ☐
   - NO ☐

   If YES, what is the age of the younger/youngest one?.....................
6. How easy or difficult is it to get employment in the salon business? [Probe]
   - Easy ☐
   - Somewhat Easy ☐
   - Somewhat difficult ☐

Difficult ☐

7. How long have you worked as a salonist?

Less than 1 Year	
More than 1 year but less than 5 Years	
Between 5-10 Years	
More than 10 Years	

Part B: KNOWLEDGE OF RISKS FROM COSMETICS

8. Please check or place a tick against any class of cosmetics and personal care products (PCPs) that you use.

**Cosmetics products and PCPs**	**Answer**
**Personal care products**	
Hair conditioner	
Hair dyes	
Nail polish	
Nail polish remover	
Face and body lotions	
Shampoos	
Hair gel/lotion	
Shaving gels and sprays	
Moisturizers	
Perfumes, cologne, body sprays	
Deodorants and antiperspirants	
Specify any other..	
**Cosmetics**	
Foundations	
Concealers	
Mascara	
Eye shadows	
Eyeliners	
Lipsticks and lip gloss	

Specify any other..	

9. Please check/tick the box that best represents your understanding of each of the following statements. (PCPs = Personal care products)

	**Correct**	**Incorrect**	**Don't know**
Exposure to cosmetics and PCPs can cause ill health			
There are protective measures I can take when using cosmetic products or personal care products			
Cosmetics and PCPs only cause harm to children			
Cosmetics and PCPs only cause harm under prolonged exposure			
Cosmetics and PCPs cause harm when used in large amounts			

10. Cosmetics and personal care products can cause harm if

	**Correct**	**Incorrect**	**Don't know**
Ingested			
Inhaled			
Skin absorption			
Injected			

11. What harm can arise from unprotected exposure to cosmetics and personal care products?
………………………………………………………………………………………………………
12. What protective measures are available for you at your work place? Please check those that apply

	Available	Not available	Don't know
Separate rooms or spaces to use for certain cosmetics products or procedures			
Mops and other resources for dealing with accidental spills			
Air vents			
Protective clothing such as gloves, aprons and face masks			
Buckets or other containers for waste disposal			

Specify any other:

Part C: RISK PERCEPTION

13. Do you think that exposure to cosmetics and personal care products used in beauty parlors can harm your health if you do not use protective measures?

Not at all likely	Not likely	Somewhat likely	Very likely	Extremely likely

14. Do you think that exposure to cosmetics and personal care products during pregnancy can harm your unborn child?

Not at all likely	Not likely	Somewhat likely	Very likely	Extremely likely

15. Do you know anyone who has suffered ill health due to exposure to cosmetics and personal care products?

YES	
NO	

If YES, please explain…………………………………………………………………………

16. Have you been using protective measures when handling cosmetics and personal care products at the beauty parlor?

Never	Rarely	Some times	Regularly	Always

17. If yes, what protective measures have you been using?...

……………………………………………………………………………………………………….

18. Do you think that using cosmetics and personal care products may have already affected you negatively?

YES	
NO	

If YES, please explain…………………………………………………………………

19. Suppose you have been taking protective measures when using cosmetic products. What do you think your chance of suffering harmful effects from use of cosmetics and personal care products is?

No chance	Very little chance	Little chance	Large chance	Very large chance

20. How concerned are you about ill health resulting from exposure to cosmetics products?

Not at all concerned	Not concerned	Slightly concerned	Concerned	Very concerned

21. When buying cosmetics, please rate which of the following issues that you pay attention to

	Never	Rarely	Occasionally	Often	Always
Price					
Manufacturer					
Ingredients					
Fragrance					
Advertisement					
Brand					
Recommendation by clients					
Recommendation by other saloonists					
Trial samples					
Celebrity endorsement					
Research on its manufacturing process including animal testing					
Other, specify:					

Part D: INTENTION TO CARRY OUT PROTECTIVE MEASURES

22. Would you carry out protective measures if this was advised?

Certainly not	Probably not	Probably yes	Certainly	Most certainly

23. Are there protective measures that you would carry out if you are advised to do so?

YES		Answer Question 24
NO		Skip to Question 25

Part E: MOTIVATING/HINDERING FACTORS

24. Why would you be willing to carry out protective measures if you were to be advised to do so? Please indicate your level of agreement with each of the statements below where 1 = Strongly Agree, 2 = Agree, 3 = Neither Agree nor Disagree, 4 = Disagree and 5 = Strongly Disagree.

**Statement**	**1**	**2**	**3**	**4**	**5**
I am often ill					
Exposure to cosmetic products can be serious					
I feel responsible for my health					
I think I am at risk of cosmetic products that can cause ill health					
I want to protect people around me from any harm arising from exposure to cosmetic products					
I trust that the protective measures help					
I will use protective measures if I am advised to					
If I do not take these measures, I may regret it later					
Other people in my environment will also carry out the measures					

Other reason, specify……………………………………………………..

25. Why would you **NOT** be willing to carry out protective measures if you were to be advised to do so? Please indicate your level of agreement with each of the statements below where 1 = Strongly Agree, 2 = Agree, 3 = Neither Agree nor Disagree, 4 = Disagree and 5 = Strongly Disagree.

**Statement**	**1**	**2**	**3**	**4**	**5**
I am never ill					
Unprotected exposure to cosmetic is not harmful					
I do not find it important					
I am not worried about my health					
I do not think I am at risk of harm from unprotected exposure to cosmetics.					
I do not think that I would transfer any ill harm or ill health I get from unprotected exposure to cosmetics to other people					
I doubt whether the protective measures help					
Takes too much effort or time					
People in my environment will also not carry out the measures					

I feel that too little information is provided about the measures					
I cannot afford protective measures					
For principle reasons (e.g. religion/anthroposophical conviction)					

Other, namely……………………………………………………………

Part F: INFORMATION NEEDS

26. What are the most important topics for which you would like to receive information at this time? Tick as many as THREE topics that you consider most important.

Topic	Answer
a. How to protect yourself from exposure to cosmetic products	
b. How to protect others from exposure to cosmetic products	
c. What protective measures are available for those using cosmetic products	
d. Which cosmetic products are safe	
e. What to do when you are exposed cosmetic products known to cause ill health	
f. Symptoms of ill health from exposure to cosmetic products	

Any others, specify…………………………………………………………………

27. Who would you like to provide you with this information? Tick those you would like to provide you with information. (more than one answer possible).

Provider of information	Answer
a. General practitioner	
b. County Public Health Service	
c. National authorities (for example, Ministry of Health)	
d. Unknown	
Other, namely……………………………………………………………	

28. How would you like to receive this information? (up to 3 answers possible)

Means of communication	Answer
a. Information meeting by the Ministry of Health	
b. Leaflets from the County Government	
c. Leaflets from the National Government	
d. Information in local newspapers	
e. Details on product inserts	
f. Information on local radio stations	
Other, namely………………………………………………….	

As we close, do you have any remarks on this questionnaire?

We thank you for your participation in the survey.
